# Ten simple rules for tackling your first mathematical models: A guide for graduate students by graduate students

**DOI:** 10.1371/journal.pcbi.1008539

**Published:** 2021-01-14

**Authors:** Korryn Bodner, Chris Brimacombe, Emily S. Chenery, Ariel Greiner, Anne M. McLeod, Stephanie R. Penk, Juan S. Vargas Soto

**Affiliations:** 1 Department of Biological Sciences, University of Toronto Scarborough, Toronto, Ontario, Canada; 2 Department of Ecology and Evolution, University of Toronto, Toronto, Ontario, Canada; 3 Department of Physical and Environmental Sciences, University of Toronto Scarborough, Toronto, Ontario, Canada; 4 Department of Biology, Memorial University of Newfoundland, St John’s, Newfoundland, Canada; Dassault Systemes BIOVIA, UNITED STATES

## Introduction

Biologists spend their time studying the natural world, seeking to understand its various patterns and the processes that give rise to them. One way of furthering our understanding of natural phenomena is through laboratory or field experiments, examining the effects of changing one, or several, variables on a measured response. Alternatively, one may conduct an observational study, collecting field data and comparing a measured response along natural gradients. A third and complementary way of understanding natural phenomena is through mathematical models. In the life sciences, more scientists are incorporating these quantitative methods into their research. Given the vast utility of mathematical models, ranging from providing qualitative predictions to helping disentangle multiple causation (see Hurford [1] for a more complete list), their increased adoption is unsurprising. However, getting started with mathematical models may be quite daunting for those with traditional biological training, as in addition to understanding new terminology (e.g., “Jacobian matrix,” “Markov chain”), one may also have to adopt a different way of thinking and master a new set of skills.

Here, we present 10 simple rules for tackling your first mathematical models. While many of these rules are applicable to basic scientific research, our discussion relates explicitly to the process of model-building within ecological and epidemiological contexts using dynamical models. However, many of the suggestions outlined below generalize beyond these disciplines and are applicable to nondynamic models such as statistical models and machine-learning algorithms. As graduate students ourselves, we have created rules we wish we had internalized before beginning our model-building journey—a guide by graduate students, for graduate students—and we hope they prove insightful for anyone seeking to begin their own adventures in mathematical modelling.

**Fig 1:**
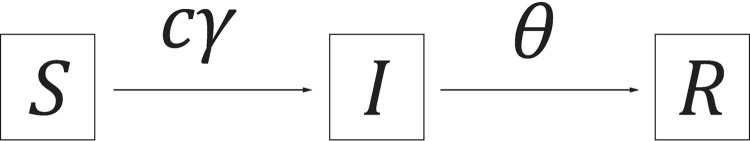
SIR conceptual diagram. Boxes represent susceptible, infected, and recovered compartments, and directed arrows represent the flow of individuals between these compartments with the rate of flow being controlled by the contact rate, *c*, the probability of infection, *γ*, and the recovery rate, *θ*.

When necessary, we illustrate our rules by referring to a susceptible-infected-recovered (SIR) model, a class of models commonly adopted for population-level disease dynamics (e.g., Influenza [2], SARS [3], and more recently, Coronavirus Disease 2019 (COVID-19) [4]). The basic SIR model is an easily accessible and tangible example of a mathematical model that should be familiar to anyone who has taken an introductory modelling class. It is a system of ordinary differential equations (ODEs) that can be represented as a conceptual diagram (Fig 1) and modelled using the following equations:

$$\frac{ds}{dt}=-c\gamma IS$$ (1)

$$\frac{dI}{dt}=c\gamma IS-\theta I$$ (2)

$$\frac{dR}{dt}=\theta I$$ (3)

where *S* is the number of susceptible individuals, *I* is the number of infected individuals, *R* is the number of recovered individuals, and *N = S+I+R* is the total population size. The transmission rate is the product of the rate of contact between individuals, *c*, and the probability of infection given contact, *γ*. Infected individuals recover at a rate *θ*, and immunity is maintained so they remain in the recovered class after infection clears. We recognize this model is simple and not representative of the more complex models that researchers must often adopt but also note that even in simple cases, translating conceptual diagrams to equations may not be trivial. However, while we empathize with the difficulties more complexity inevitably brings (and provide advice on how to handle some of these complexities in the rules below), the simplicity of the above system allows for easy illustration of our fundamental points. Through simplicity, we hope to communicate knowledge that is useful even when building more complex models.

### Rule 1: Know your question

“All models are wrong, some are useful” is a common aphorism, generally attributed to statistician George Box, but determining which models are useful is dependent upon the question being asked. The practice of clearly defining a research question is often drilled into aspiring researchers in the context of selecting an appropriate research design, interpreting statistical results, or when outlining a research paper. Similarly, the practice of defining a clear research question is important for mathematical models as their results are only as interesting as the questions that motivate them [5]. The question defines the model’s main purpose and, in all cases, should extend past the goal of merely building a model for a system (the question can even answer whether a model is even necessary). Ultimately, the model should provide an answer to the research question that has been proposed.

When the research question is used to inform the purpose of the model, it also informs the model’s structure. Given that models can be modified in countless ways, providing a purpose to the model can highlight why certain aspects of reality were included in the structure while others were ignored [6]. For example, when deciding whether we should adopt a more realistic model (i.e., add more complexity), we can ask whether we are trying to inform general theory or whether we are trying to model a response in a specific system. For example, perhaps we are trying to predict how fast an epidemic will grow based on different age-dependent mixing patterns. In this case, we may wish to adapt our basic SIR model to have age-structured compartments if we suspect this factor is important for the disease dynamics. However, if we are exploring a different question, such as how stochasticity influences general SIR dynamics, the age-structured approach would likely be unnecessary. We suggest that one of the first steps in any modelling journey is to choose the processes most relevant to your question (i.e., your hypothesis) and the direct and indirect causal relationships among them: Are the relationships linear, nonlinear, additive, or multiplicative? This challenge can be aided with a good literature review. Depending on your model purpose, you may also need to spend extra time getting to know your system and/or the data before progressing forward. Indeed, the more background knowledge acquired when forming your research question, the more informed your decision-making when selecting the structure, parameters, and data for your model.

### Rule 2: Define multiple appropriate models

**Fig 2:**
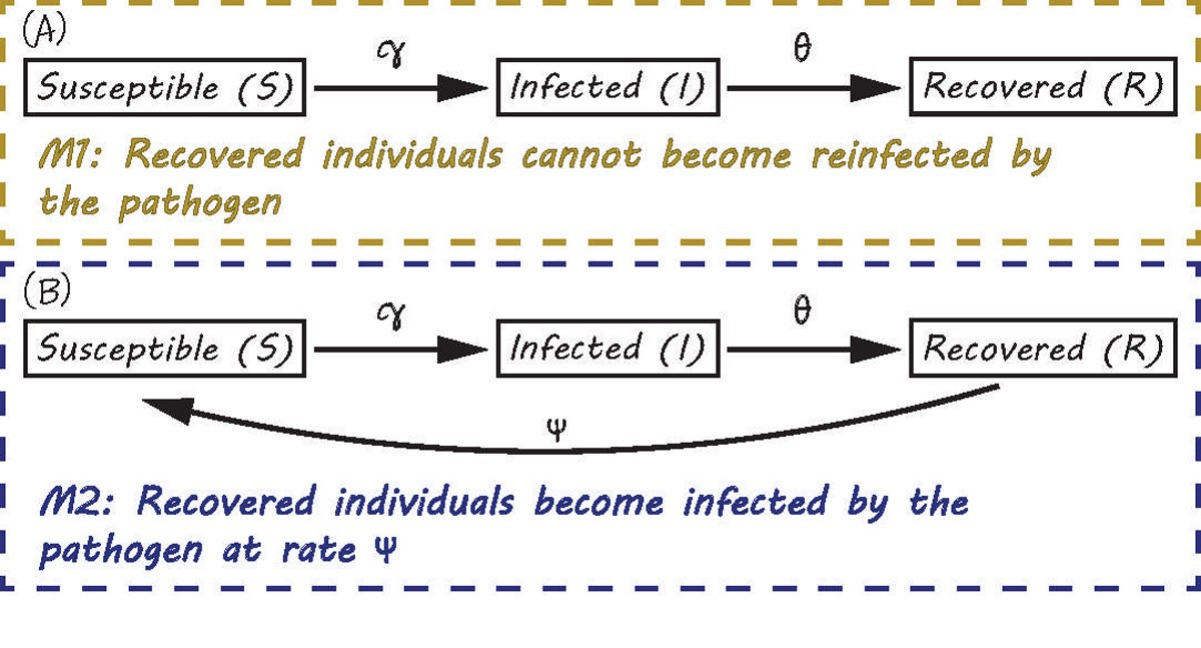
Conceptual diagrams for 2 different SIR type models. (A) A susceptible/infected/recovered model where individuals remain immune (gold) and (B) a susceptible/infected/recovered model where individuals can become susceptible again (blue). Arrows indicate the direction of movement between compartments, *c* is the contact rate, *γ* is the infection rate given contact, and *θ* is the recovery rate. The text below each conceptual model are the hypotheses (*H1* and *H2*) that represent the differences between these 2 SIR models.

Natural phenomena are complicated to study and often impossible to model in their entirety. We are often unsure about the variables or processes required to fully answer our research question(s). For example, we may not know how the possibility of reinfection influences the dynamics of a disease system. In cases such as these, our advice is to produce and sketch out a set of candidate models that consider alternative terms/variables which may be relevant for the phenomena under investigation. As in Fig 2, we construct 2 models, one that includes the ability for recovered individuals to become infected again, and one that does not. When creating multiple models, our general objective may be to explore how different processes, inputs, or drivers affect an outcome of interest or it may be to find a model or models that best explain a given set of data for an outcome of interest. In our example, if the objective is to determine whether reinfection plays an important role in explaining the patterns of a disease, we can test our SIR candidate models using incidence data to determine which model receives the most empirical support. Here we consider our candidate models to be alternative hypotheses, where the candidate model with the least support is discarded. While our perspective of models as hypotheses is a view shared by researchers such as Hilborn and Mangel [7], and Penk and colleagues [8], please note that others such as Oreskes and colleagues [9] believe that models are not subject to proof and hence disagree with this notion. We encourage modellers who are interested in this debate to read the provided citations.

Finally, we recognize that time and resource constraints may limit the ability to build multiple models simultaneously; however, even writing down alternative models on paper can be helpful as you can always revisit them if your primary model does not perform as expected. Of course, some candidate models may not be feasible or relevant for your system, but by engaging in the activity of creating multiple models, you will likely have a broader perspective of the potential factors and processes that fundamentally shape your system.

### Rule 3: Determine the skills you will need (and how to get them)

Equipping yourself with the necessary analytical tools that form the basis of all quantitative techniques is essential. As Darwin said, those that have knowledge of mathematics seem to be endowed with an extra sense [10], and having a background in calculus, linear algebra, and statistics can go a long way. Thus, make it a habit to set time for yourself to learn these mathematical skills, and do not treat all your methods like a black box. For instance, if you plan to use ODEs, consider brushing up on your calculus, e.g., using Stewart [11]. If you are working with a system of ODEs, also read up on linear algebra, e.g., using Poole [12]. Some universities also offer specialized math biology courses that combine topics from different math courses to teach the essentials of mathematical modelling. Taking these courses can help save time, and if they are not available, their syllabi can help focus your studying. Also note that while narrowing down a useful skillset in the early stages of model-building will likely spare you from some future headaches, as you progress in your project, it is inevitable that new skills will be required. Therefore, we advise you to check in at different stages of your modelling journey to assess the skills that would be most relevant for your next steps and how best to acquire them. Hopefully, these decisions can also be made with the help of your supervisor and/or a modelling mentor. Building these extra skills can at first seem daunting but think of it as an investment that will pay dividends in improving your future modelling work.

When first attempting to tackle a specific problem, find relevant research that accomplishes the same tasks and determine if you understand the processes and techniques that are used in that study. If you do, then you can implement similar techniques and methods, and perhaps introduce new methods. If not, then determine which tools you need to add to your toolbox. For instance, if the problem involves a system of ODEs (e.g., SIR models, see above), can you use existing symbolic software (e.g., Maple, Matlab, Mathematica) to determine the systems dynamics via a general solution, or is the complexity too great that you will need to create simulations to infer the dynamics? Figuring out questions like these is key to understanding what skills you will need to work with the model you develop. While there is a time and a place for involving collaborators to help facilitate methods that are beyond your current reach, we strongly advocate that you approach any potential collaborator only after you have gained some knowledge of the methods first. Understanding the methodology, or at least its foundation, is not only crucial for making a fruitful collaboration, but also important for your development as a scientist.

### Rule 4: Do not reinvent the wheel

While we encourage a thorough understanding of the methods researchers employ, we simultaneously discourage unnecessary effort redoing work that has already been done. Preventing duplication can be ensured by a thorough review of the literature (but note that reproducing original model results can advance your knowledge of how a model functions and lead to new insights in the system). Often, we are working from established theory that provides an existing framework that can be applied to different systems. Adapting these frameworks can help advance your own research while also saving precious time. When digging through articles, bear in mind that most modelling frameworks are not system-specific. Do not be discouraged if you cannot immediately find a model in your field, as the perfect model for your question may have been applied in a different system or be published only as a conceptual model. These models are still useful! Also, do not be shy about reaching out to authors of models that you think may be applicable to your system. Finally, remember that you can be critical of what you find, as some models can be deceptively simple or involve assumptions that you are not comfortable making. You should not reinvent the wheel, but you can always strive to build a better one.

### Rule 5: Study and apply good coding practices

The modelling process will inevitably require some degree of programming, and this can quickly become a challenge for some biologists. However, learning to program in languages commonly adopted by the scientific community (e.g., R, Python) can increase the transparency, accessibility, and reproducibility of your models. Even if you only wish to adopt preprogrammed models, you will likely still need to create code of your own that reads in data, applies functions from a collection of packages to analyze the data, and creates some visual output. Programming can be highly rewarding—you are creating something after all—but it can also be one of the most frustrating parts of your research. What follows are 3 suggestions to avoid some of the frustration.

Organization is key, both in your workflow and your written code. Take advantage of existing software and tools that facilitate keeping things organized. For example, computational notebooks like Jupyter notebooks or R-Markdown documents allow you to combine text, commands, and outputs in an easily readable and shareable format. Version control software like Git makes it simple to both keep track of changes as well as to safely explore different model variants via branches without worrying that the original model has been altered. Additionally, integrating with hosting services such as Github allows you to keep your changes safely stored in the cloud. For more details on learning to program, creating reproducible research, programming with Jupyter notebooks, and using Git and Github, see the 10 simple rules by Carey and Papin [13], Sandve and colleagues [14], Rule and colleagues [15], and Perez-Riverol and colleagues [16], respectively.

**Fig 3:**
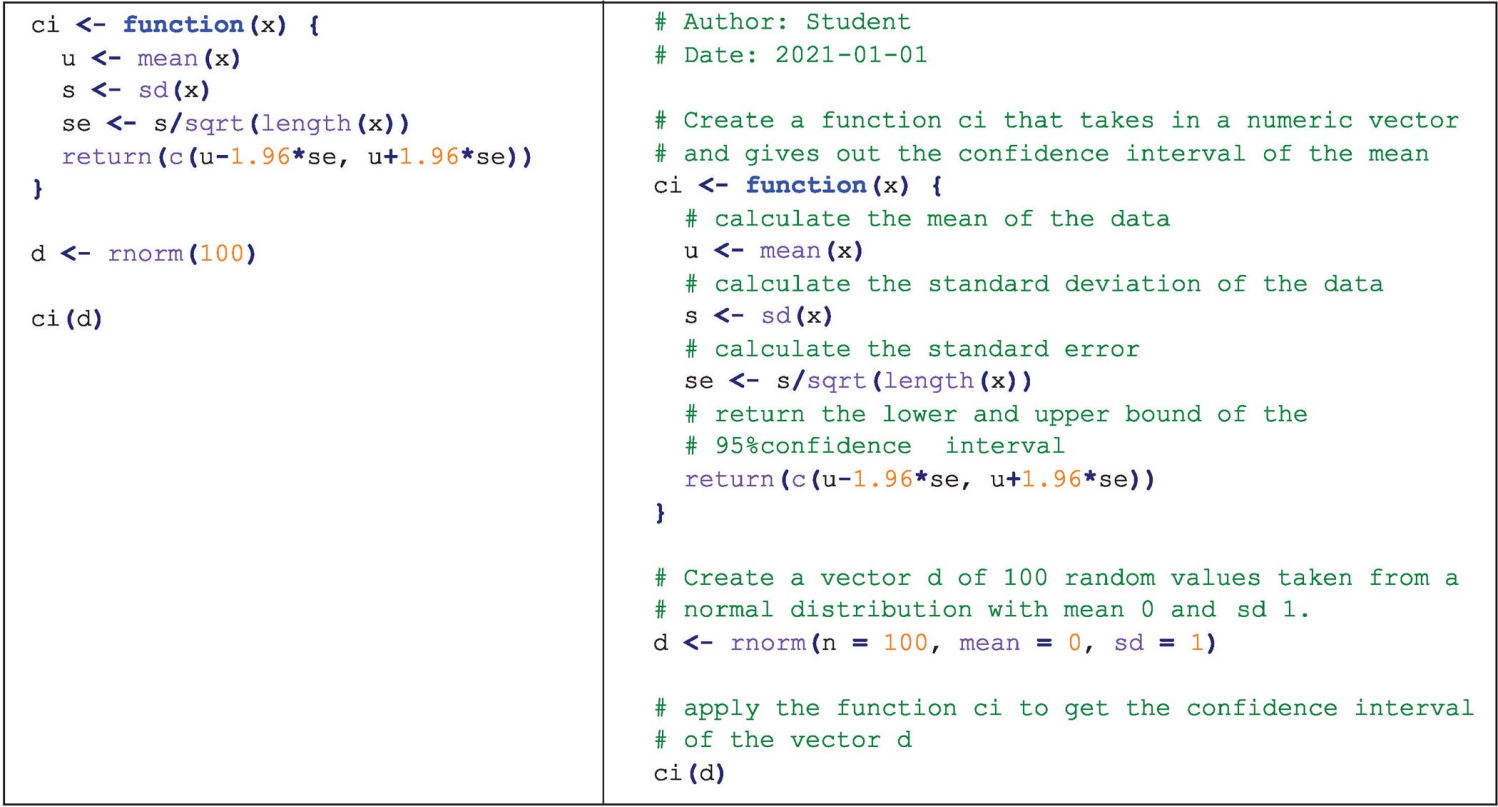
Good coding practice. Two functionally identical codes in R [17] can look very different without comments (left) and with descriptive comments (right). Writing detailed comments will help you and others understand, adapt, and use your code.

Comment your code and comment it well (see Fig 3). These comments can be the pseudocode you have written on paper prior to coding. Assume that when you revisit your code weeks, months, or years later, you will have forgotten most of what you did and why you did it. Good commenting can also help others read and use your code, making it a critical part of increasing scientific transparency. It is always good practice to write your comments before you write the code, explaining what the code should do. When coding a function, include a description of its inputs and outputs. We also encourage you to publish your commented model code in repositories such that they are easily accessible to others—not only to get useful feedback for yourself but to provide the modelling foundation for others to build on.

When writing long code, test portions of it separately. If you are writing code that will require a lot of processing power or memory to run, use a simple example first, both to estimate how long the project will take, and to avoid waiting 12 hours to see if it works. Additionally, when writing code, try to avoid too many packages and “tricks” as it can make your code more difficult to understand. Do not be afraid of writing 2 separate functions if it will make your code more intuitive. As with writing, your skill as a writer is not dependent on your ability to use big words, but instead about making sure your reader understands what you are trying to communicate.

### Rule 6: Sweat the “right” small stuff

By “sweat the ‘right’ small stuff,” we mean considering the details and assumptions that can potentially make or break a mathematical model. A good start would be to ensure your model follows the rules of mass and energy conservation. In a closed system, mass and energy cannot be created nor destroyed, and thus, the left side of the mathematical equation must equal the right under all circumstances. For example, in Eq 2, if the number of susceptible individuals decreases due to infection, we must include a negative term in this equation (−*cγIS*) to indicate that loss and its conjugate (+*cγIS*) to the infected individuals equation, Eq 3, to represent that gain. Similarly, units of all terms must also be balanced on both sides of the equation. For example, if we wish to add or subtract 2 values, we must ensure their units are equivalent (e.g., cannot add day ^−1^ and year ^−1^). Simple oversights in units can lead to major setbacks and create bizarre dynamics, so it is worth taking the time to ensure the units match up.

Modellers should also consider the fundamental boundary conditions of each parameter to determine if there are some values that are illogical. Logical constraints and boundaries can be developed for each parameter using prior knowledge and assumptions (e.g., Huntley [18]). For example, when considering an SIR model, there are 2 parameters that comprise the transmission rate—the contact rate, *c*, and the probability of infection given contact, *γ*. Using our intuition, we can establish some basic rules: (1) the contact rate cannot be negative; (2) the number of susceptible, infected, and recovered individuals cannot be below 0; and (3) the probability of infection given contact must fall between 0 and 1. Keeping these in mind as you test your model’s dynamics can alert you to problems in your model’s structure. Finally, simulating your model is an excellent method to obtain more reasonable bounds for inputs and parameters and ensure behavior is as expected. See Otto and Day [5] for more information on the “basic ingredients” of model-building.

### Rule 7: Simulate, simulate, simulate

**Fig 4:**
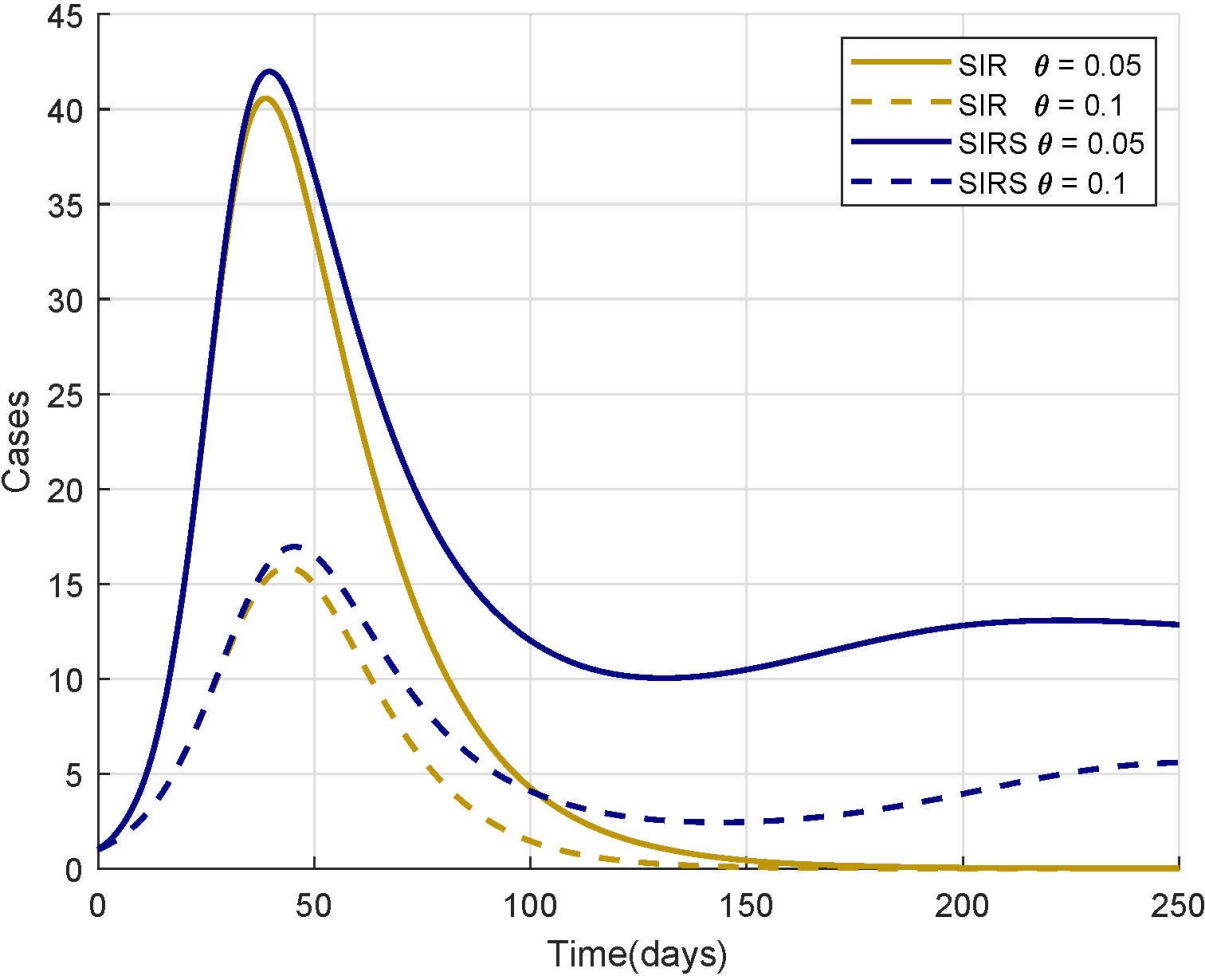
Simulated model output using 2 different model structures and 2 different parameter values. Gold lines represent the SIR structure (Fig 2A) where lifelong immunity of individuals is inferred after infection, and blue lines represent an SIRS structure (Fig 2B) where immunity is lost over time. The solid lines represent model dynamics assuming a recovery rate (*θ*) of 0.05, while dotted lines represent dynamics assuming a recovery rate of 0.1. All model runs assume a transmission rate, *cγ*, of 0.2 and an immunity loss rate, *ψ*, of 0.01. By using simulations, we can explore how different processes and rates change the system’s dynamics and furthermore determine at what point in time these differences are detectable. SIR, Susceptible-Infected-Recovered; SIRS, Susceptible-Infected-Recovered-Susceptible.

Even though there is a lot to be learned from analyzing simple models and their general solutions, modelling a complex world sometimes requires complex equations. Unfortunately, the cost of this complexity is often the loss of general solutions [19]. Instead, many biologists must calculate a numerical solution, an approximate solution, and simulate the dynamics of these models [20]. Simulations allow us to explore model behavior, given different structures, initial conditions, and parameters (Fig 4). Importantly, they allow us to understand the dynamics of complex systems that may otherwise not be ethical, feasible, or economically viable to explore in natural systems [21].

One common method of exploring the dynamics of complex systems is through sensitivity analysis (SA). We can use this simulation-based technique to ascertain how changes in parameters and initial conditions will influence the behavior of a system. For example, if simulated model outputs remain relatively similar despite large changes in a parameter value, we can expect the natural system represented by that model to be robust to similar perturbations. If instead, simulations are very sensitive to parameter values, we can expect the natural system to be sensitive to its variation. Here in Fig 4, we can see that both SIR models are very sensitive to the recovery rate parameter (*θ*) suggesting that the natural system would also be sensitive to individuals’ recovery rates. We can therefore use SA to help inform which parameters are most important and to determine which are distinguishable (i.e., identifiable). Additionally, if observed system data are available, we can use SA to help us establish what are the reasonable boundaries for our initial conditions and parameters. When adopting SA, we can either vary parameters or initial conditions one at a time (local sensitivity) or preferably, vary multiple of them in tandem (global sensitivity). We recognize this topic may be overwhelming to those new to modelling so we recommend reading Marino and colleagues [22] and Saltelli and colleagues [23] for details on implementing different SA methods.

Simulations are also a useful tool for testing how accurately different model fitting approaches (e.g., Maximum Likelihood Estimation versus Bayesian Estimation) can recover parameters. Given that we know the parameter values for simulated model outputs (i.e., simulated data), we can properly evaluate the fitting procedures of methods when used on that simulated data. If your fitting approach cannot even recover simulated data with known parameters, it is highly unlikely your procedure will be successful given real, noisy data. If a procedure performs well under these conditions, try refitting your model to simulated data that more closely resembles your own dataset (i.e., imperfect data). If you know that there was limited sampling and/or imprecise tools used to collect your data, consider adding noise, reducing sample sizes, and adding temporal and spatial gaps to see if the fitting procedure continues to return reasonably correct estimates. Remember, even if your fitting procedures continue to perform well given these additional complexities, issues may still arise when fitting to empirical data. Models are approximations and consequently their simulations are imperfect representations of your measured outcome of interest. However, by evaluating procedures on perfectly known imperfect data, we are one step closer to having a fitting procedure that works for us even when it seems like our data are against us.

### Rule 8: Expect model fitting to be a lengthy, arduous but creative task

Model fitting requires an understanding of both the assumptions and limitations of your model, as well as the specifics of the data to be used in the fitting. The latter can be challenging, particularly if you did not collect the data yourself, as there may be additional uncertainties regarding the sampling procedure, or the variables being measured. For example, the incidence data commonly adopted to fit SIR models often contain biases related to underreporting, selective reporting, and reporting delays [24]. Taking the time to understand the nuances of the data is critical to prevent mismatches between the model and the data. In a bad case, a mismatch may lead to a poor-fitting model. In the worst case, a model may appear well-fit, but will lead to incorrect inferences and predictions.

Model fitting, like all aspects of modelling, is easier with the appropriate set of skills (see Rule 2). In particular, being proficient at constructing and analyzing mathematical models does not mean you are prepared to fit them. Fitting models typically requires additional in-depth statistical knowledge related to the characteristics of probability distributions, deriving statistical moments, and selecting appropriate optimization procedures. Luckily, a substantial portion of this knowledge can be gleaned from textbooks and methods-based research articles. These resources can range from covering basic model fitting, such as determining an appropriate distribution for your data and constructing a likelihood for that distribution (e.g., Hilborn and Mangel [7]), to more advanced topics, such as accounting for uncertainties in parameters, inputs, and structures during model fitting (e.g., Dietze [25]). We find these sources among others (e.g., Hobbs and Hooten [26] for Bayesian methods; e.g., Adams and colleagues [27] for fitting noisy and sparse datasets; e.g., Sirén and colleagues [28] for fitting individual-based models; and Williams and Kendall [29] for multiobject optimization—to name a few) are not only useful when starting to fit your first models, but are also useful when switching from one technique or model to another.

After you have learned about your data and brushed up on your statistical knowledge, you may still run into issues when model fitting. If you are like us, you will have incomplete data, small sample sizes, and strange data idiosyncrasies that do not seem to be replicated anywhere else. At this point, we suggest you be explorative in the resources you use and accept that you may have to combine multiple techniques and/or data sources before it is feasible to achieve an adequate model fit (see Rosenbaum and colleagues [30] for parameter estimation with multiple datasets). Evaluating the strength of different techniques can be aided by using simulated data to test these techniques, while SA can be used to identify insensitive parameters which can often be ignored in the fitting process (see Rule 7).

Model accuracy is an important metric but “good” models are also precise (i.e., reliable). During model fitting, to make models more reliable, the uncertainties in their inputs, drivers, parameters, and structures, arising due to natural variability (i.e., aleatory uncertainty) or imperfect knowledge (i.e., epistemic uncertainty), should be identified, accounted for, and reduced where feasible [31]. Accounting for uncertainty may entail measurements of uncertainties being propagated through a model (a simple example being a confidence interval), while reducing uncertainty may require building new models or acquiring additional data that minimize the prioritized uncertainties (see Dietze [25] and Tsigkinopoulou and colleagues [32] for a more thorough review on the topic). Just remember that although the steps outlined in this rule may take a while to complete, when you do achieve a well-fitted reliable model, it is truly something to be celebrated.

### Rule 9: Give yourself time (and then add more)

Experienced modellers know that it often takes considerable time to build a model and that even more time may be required when fitting to real data. However, the pervasive caricature of modelling as being “a few lines of code here and there” or “a couple of equations” can lead graduate students to hold unrealistic expectations of how long finishing a model may take (or when to consider a model “finished”). Given the multiple considerations that go into selecting and implementing models (see previous rules), it should be unsurprising that the modelling process may take weeks, months, or even years. Remembering that a published model is the final product of long and hard work may help reduce some of your time-based anxieties. In reality, the finished product is just the tip of the iceberg and often unseen is the set of failed or alternative models providing its foundation. Note that taking time early on to establish what is “good enough” given your objective, and to instill good modelling practices, such as developing multiple models, simulating your models, and creating well-documented code, can save you considerable time and stress.

### Rule 10: Care about the process, not just the endpoint

As a graduate student, hours of labor coupled with relative inexperience may lead to an unwillingness to change to a new model later down the line. But being married to one model can restrict its efficacy, or worse, lead to incorrect conclusions. Early planning may mitigate some modelling problems, but many issues will only become apparent as time goes on. For example, perhaps model parameters cannot be estimated as you previously thought, or assumptions made during model formulation have since proven false. Modelling is a dynamic process, and some steps will need to be revisited many times as you correct, refine, and improve your model. It is also important to bear in mind that the process of model-building is worth the effort. The process of translating biological dynamics into mathematical equations typically forces us to question our assumptions, while a misspecified model often leads to novel insights. While we may wish we had the option to skip to a final finished product, in the words of Drake, “sometimes it’s the journey that teaches you a lot about your destination”.

### Conclusion

There is no such thing as a failed model. With every new error message or wonky output, we learn something useful about modelling (mostly begrudgingly) and, if we are lucky, perhaps also about the study system. It is easy to cave in to the ever-present pressure to perform, but as graduate students, we are still learning. Luckily, you are likely surrounded by other graduate students, often facing similar challenges who can be an invaluable resource for learning and support. Finally, remember that it does not matter if this was your first or your 100th mathematical model, challenges will always present themselves. However, with practice and determination, you will become more skilled at overcoming them, allowing you to grow and take on even greater challenges.
